# Liver cirrhosis and hepatocellular carcinoma: chronological decoupling of biochemical clearance and mechanical regeneration signals a systems biology hypothesis on programed deconstruction failure

**DOI:** 10.3389/fonc.2026.1799039

**Published:** 2026-05-04

**Authors:** Yanming Li

**Affiliations:** Department of Hepatobiliary Surgery, Qilu Hospital of Shandong University (Dezhou Hospital), Dezhou, Shandong, China

**Keywords:** chronological decoupling, hepatocellular carcinoma, liver cirrhosis, LOXL2, macrophage polarization, piezo1-YAP Axis

## Abstract

Traditional hepatology conceptualizes liver cirrhosis as the linear accumulation of extracellular matrix (ECM). However, the stagnation of fibrosis reversal and the persistent risk of hepatocellular carcinoma (HCC) despite eradication of the etiology remain significant clinical challenges. We propose the “Chronological Decoupling” hypothesis: cirrhosis arises from a specific temporal mismatch (\Delta t > physiological resolution window of 7–14 days) between Signal A (biochemical clearance, defined as >50% shift of macrophages to TREM2+ restorative phenotype with MMP-9/MMP-13 secretion) and Signal B (mechanical regeneration, defined as Piezo1-mediated YAP nuclear translocation >50% in hepatocytes under lateral crowding pressure). In chronic injury, Signal B is triggered prematurely before Signal A completes, producing “Chaotic Deconstruction” quantifiable as increased ECM fragment heterogeneity, secondary inflammation markers, and covalent cross-linking via LOXL2 . This creates an immune-excluded oncogenic niche. We shift therapeutic focus from continuous anti-fibrotics to “Protocol Resynchronization,” a biomarker-timed sequential therapy.

## 1 Introduction: the evolutionary compromise of liver containment

From an evolutionary perspective, the liver serves not only as a metabolic hub but also as a critical immune filter (1, 2). Facing chronic injury (e.g., MASLD, HBV), the liver must maintain equilibrium between organ function preservation and prevention of systemic infection. Consequently, a regional containment strategy has emerged, wherein activation of hepatic stellate cells (HSCs) forms an ECM barrier to localize damage (3, 4). Although advantageous in the acute phase, this strategy depends on an “ordered withdrawal” following injury resolution (5–7). Physiologically, ECM disassembly requires a strict temporal sequence: subsidence of inflammation (Signal A) followed by protease-driven (MMPs) matrix degradation (8, 9).In cirrhosis, this containment-deconstruction cycle is disrupted. While sharing conceptual overlap with existing paradigms — dysregulated wound healing (1), the fibrosis-cancer axis (10, 11), and the “irreversible fibrosis threshold” model (12) — the Chronological Decouplinghypothesis introduces a distinct mechanistic advance. It identifies the temporal order between biochemical clearance (Signal A) and mechanical regeneration (Signal B) as the critical “AND gate” failure, even when total ECM load remains below irreversible thresholds. This decoupling produces self-reinforcing chaotic states through premature mechanical disruption, providing a novel explanation for post-etiology reversal failure and HCC risk that prior frameworks do not fully capture (13–15).

## 2 Core hypothesis: the “and gate” logic of deconstruction

**Figure 1:**
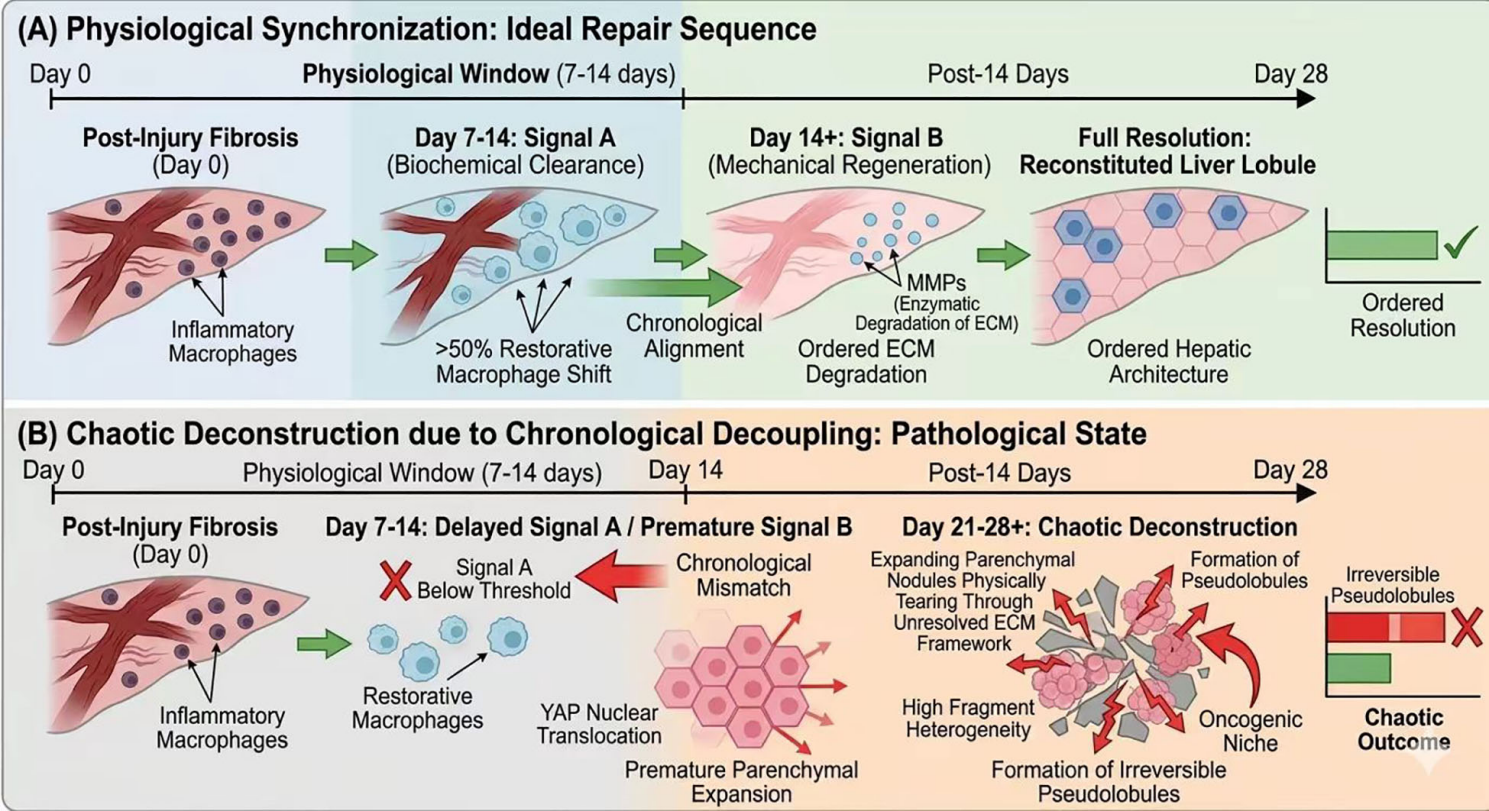
Temporal dynamics of liver fibrosis resolution. **(A)** Physiological Synchronization: Ideal Repair Sequence. Biochemical clearance (Signal A: >50% shift to restorative TREM2+ macrophages secreting MMP-9/MMP-13) precedes mechanical regeneration (Signal B: Piezo1-mediated YAP nuclear translocation) within the physiological window (7–14 days), leading to ordered ECM degradation and full restoration of hepatic architecture. **(B)** Chaotic Deconstruction due to Chronological Decoupling. Premature activation of Signal B (YAP nuclear translocation) before Signal A completion (Delta t > 7–14 days) results in mechanical tearing of unresolved ECM, high fragment heterogeneity, pseudolobule formation, and establishment of an immune-excluded oncogenic niche.

Successful reversal requires coordinated passage through a biochemical “AND gate”: both Signal A and Signal B must align temporally within the physiological resolution window (**Figure 1**).

### 2.1 Signal A: biochemical clearance and macrophage polarization

Signal A is operationally defined as the transition of proinflammatory macrophages to a restorative (TREM2+) phenotype, quantifiable by flow cytometry or spatial transcriptomics showing >50% TREM2+ shift (16, 17), accompanied by secretion of MMP-9 and MMP-13 and downregulation of profibrotic cytokines (TNF-\alpha, IL-1$\beta$ below defined thresholds) (6, 18). This halts profibrotic signaling and initiates enzymatic ECM degradation (9, 19).

### 2.2 Signal B: the piezo1-YAP axis of mechanotransduction

Signal B originates from physical expansion of parenchymal cells. As hepatocytes enter compensatory proliferation, lateral crowding activates the mechanosensitive ion channel Piezo1 (20, 21).

Biochemical Cascade: Piezo1 opening triggers local Ca^{2+} influx, subsequently activating calpain to relieve inhibition of the YAP/TAZ complex (22, 23).

Effect: Nuclear translocation of YAP leads to TEAD-binding and growth factor upregulation (22, 24).

Piezo1/YAP is prioritized over other mechanosensors (integrins, TGF-\beta-SMAD, \beta-catenin) because it directly transduces lateral crowding pressure in regenerating hepatocytes independently of initial ECM stiffness or ligand binding (20, 21, 25). Integrins require ECM ligation, TGF-\beta-SMAD is downstream of matrix stiffness, and \beta-catenin primarily regulates proliferation via Wnt rather than acute mechanosensing (22, 23). Supporting evidence shows specific Piezo1 upregulation in regenerative hepatocytes during fibrosis, positioning it as the dominant initiator of premature Signal B in decoupling scenarios (20, 21, 25, 26). In physiological sequence, this mechanical pressure guides hepatocyte repositioning only after ECM softening.

### 2.3 Chronological decoupling and chaotic deconstruction

This order is disrupted in chronic pathologies. Owing to persistent metabolic stress, hepatocytes initiate regeneration prematurely (Signal B leads, measurable as YAP nuclear translocation preceding TREM2+ shift by >7–14 days (25, 27)), while the ECM remains highly inflamed (Signal A lags (3, 19)).

Chronological Decoupling is defined as \Delta t > physiological resolution window between full Signal A completion and Signal B onset. Chaotic Deconstruction is defined as mechanically driven ECM disruption (quantifiable by increased fragment size heterogeneity via digital pathology (28) and elevated secondary inflammation markers) rather than ordered protease-mediated degradation. This leads to expanding nodules physically tearing through old ECM frameworks, inducing secondary inflammation and forming irreversible fibrous fragments (11, 13).

## 3 The “chemical lock”: LOXL2 and structural solidification

A critical determinant of deconstruction failure is LOXL2. During active inflammation, oxidative stress upregulates LOXL2 secretion, which catalyzes covalent cross-linking of lysine residues in collagen (23, 29).

The Locking Mechanism: During decoupling, ECM fragments undergoing chaotic deconstruction are prematurely “locked” by LOXL2 (measurable as increased collagen cross-link density (12, 29)).

Clinical Implications: This explains the limited efficacy of LOXL2 inhibitors like Simtuzumab in advanced cirrhosis (8). Prior trials failed because inhibitors were administered after pre-existing locks had already solidified fragmented structures; blocking *de novo*cross-linking cannot dismantle established covalent bonds (12, 30).

**Figure 2:**
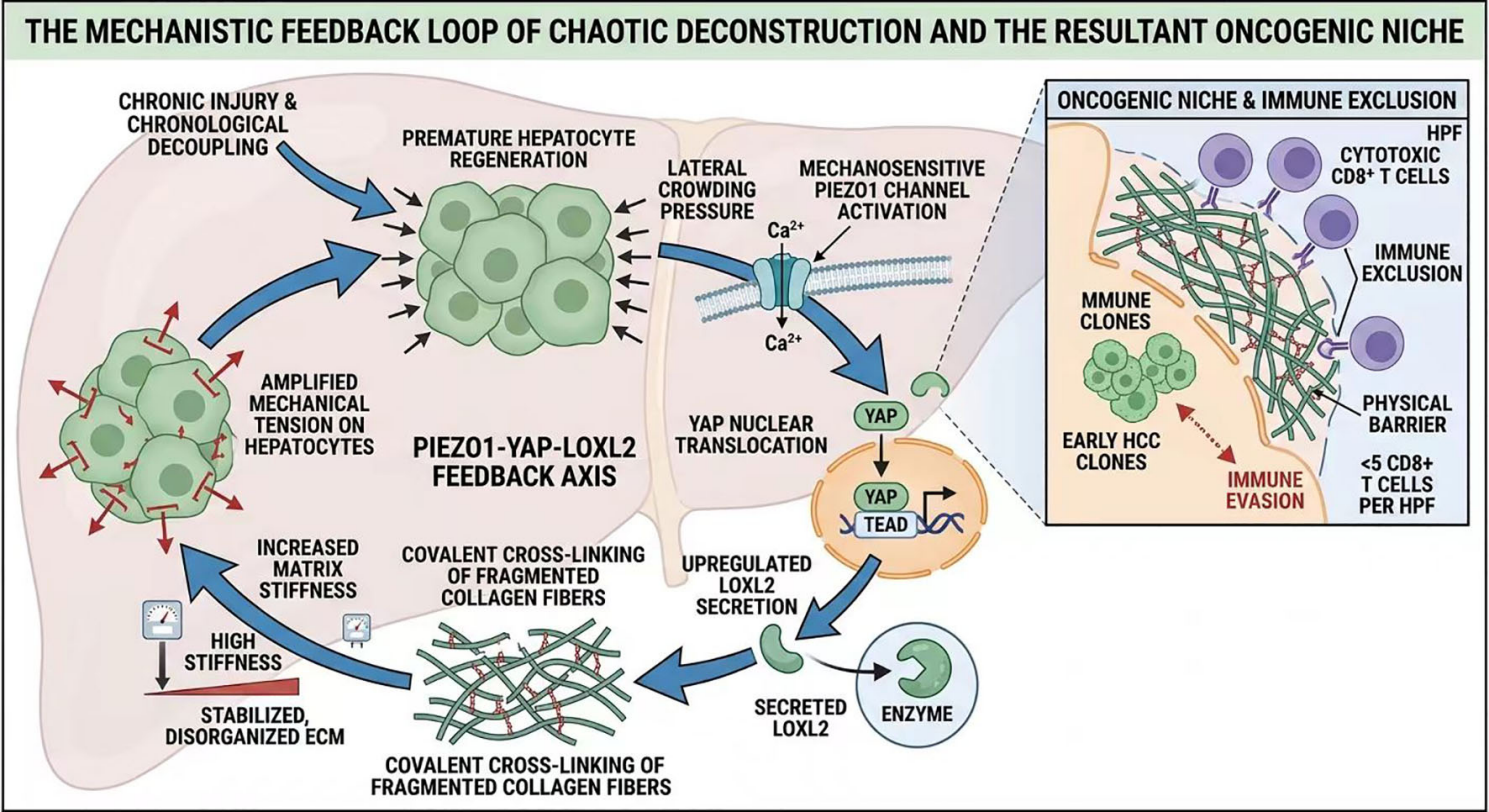
The mechanistic feedback loop of chaotic deconstruction and the resultant oncogenic niche. Mechanical tension from premature hepatocyte regeneration activates the mechanosensitive Piezo1 channel, triggering Ca²^+^ influx and YAP nuclear translocation. Nuclear YAP-TEAD binding upregulates LOXL2 secretion, which catalyzes covalent cross-linking of fragmented collagen fibers, increasing matrix stiffness and amplifying mechanical tension on hepatocytes—forming a self-reinforcing Piezo1-YAP-LOXL2 feedback axis. This chaotic deconstruction stabilizes a disorganized ECM and establishes an immune-excluded oncogenic niche characterized by physical barriers and <5 CD8^+^ T cells per high-power field (HPF), promoting early HCC clone survival and immune evasion.

Our Protocol Resynchronization overcomes these limitations through timed sequencing: biochemical priming (Phase 1) first enables endogenous MMP activity and partial matrix softening (6, 16), allowing subsequent LOXL2 inhibition (Phase 2) to prevent new locks while existing fragments are actively degraded (31, 32). This restores reversibility beyond monotherapy (**Figure 2**).

## 4 HCC pathogenesis: the mechanically-induced oncogenic niche

Chronological decoupling generates a microenvironmental state conducive to hepatocarcinogenesis:

Genomic Instability: In high-pressure irregular matrices, hepatocytes undergo nuclear envelope rupture during mitosis, increasing aneuploidy and mutations (13, 15).

Immune Exclusion: Operationally defined as physical compartmentalization of the cirrhotic niche, where fragmented fibrotic septa act as barriers preventing cytotoxic CD8+ T cell infiltration into hepatocyte nodules or HCC foci (10, 33). This is quantifiable by multiplex immunohistochemistry or digital pathology showing <5 CD8+ T cells per high-power field (HPF) within parenchymal compartments versus periseptal areas (17, 28, 33, 34), creating the characteristic immune-excluded phenotype and promoting immune evasion.

## 5 Clinical translation: protocol resynchronization therapy

We advocate shifting from “continuous dosing” to biomarker-guided “chronological synchronization” (35–38):

Phase 1: Biochemical Priming Use TREM2 agonists until >50% macrophage repolarization (Signal A dominance) to initiate matrix softening (6, 16).

Phase 2: Matrix Unlocking Introduce LOXL2 inhibitors only after macrophage polarization to maximize MMP efficiency and dismantle existing locks (30–32).

Phase 3: Guided Regeneration Use FGF21 or HGF analogs to direct hepatocyte expansion within a now-pliant environment (39, 40).

## Data Availability

The original contributions presented in the study are included in the article/supplementary material. Further inquiries can be directed to the corresponding author.
